# Mitigation of Cellular and Bacterial Adhesion on Laser Modified Poly (2-Methacryloyloxyethyl Phosphorylcholine)/Polydimethylsiloxane Surface

**DOI:** 10.3390/nano13010064

**Published:** 2022-12-23

**Authors:** Simona Nistorescu, Madalina Icriverzi, Paula Florian, Anca Bonciu, Valentina Marascu, Nicoleta Dumitrescu, Gratiela Gradisteanu Pircalabioru, Laurentiu Rusen, Alexandra Mocanu, Anca Roseanu, Anisoara Cimpean, Florin Grama, Valentina Dinca, Daniel A. Cristian

**Affiliations:** 1 National Institute for Laser, Plasma and Radiation Physics, 077125 Magurele, Romania; 2 Faculty of Biology, University of Bucharest, 050095 Bucharest, Romania; 3 Institute for Biochemistry of Romanian Academy, 060031 Bucharest, Romania; 4 Research Institute of the University of Bucharest, 050095 Bucharest, Romania; 5 Faculty of Chemical Engineering and Biotechnologies, University Politehnica of Bucharest, 011061 Bucharest, Romania; 6 Surgical Department, “Carol Davila” University of Medicine and Pharmacy, 030171 Bucharest, Romania

**Keywords:** MAPLE, zwitterionic coating, cellular response

## Abstract

Nowadays, using polymers with specific characteristics to coat the surface of a device to prevent undesired biological responses can represent an optimal strategy for developing new and more efficient implants for biomedical applications. Among them, zwitterionic phosphorylcholine-based polymers are of interest due to their properties to resist cell and bacterial adhesion. In this work, the Matrix-Assisted Laser Evaporation (MAPLE) technique was investigated as a new approach for functionalising Polydimethylsiloxane (PDMS) surfaces with zwitterionic poly(2-Methacryloyloxyethyl-Phosphorylcholine) (pMPC) polymer. Evaluation of the physical–chemical properties of the new coatings revealed that the technique proposed has the advantage of achieving uniform and homogeneous stable moderate hydrophilic pMPC thin layers onto hydrophobic PDMS without any pre-treatment, therefore avoiding the major disadvantage of hydrophobicity recovery. The capacity of modified PDMS surfaces to reduce bacterial adhesion and biofilm formation was tested for Gram-positive bacteria, *Staphylococcus aureus* (*S. aureus*), and Gram-negative bacteria, *Escherichia coli* (*E. coli*). Cell adhesion, proliferation and morphology of human THP-1 differentiated macrophages and human normal CCD-1070Sk fibroblasts on the different surfaces were also assessed. Biological in vitro investigation revealed a significantly reduced adherence on PDMS–pMPC of both *E. coli* (from 29 × 10 ^6^ to 3 × 10^2^ CFU/mL) and *S. aureus* (from 29 × 10^6^ to 3 × 10^2^ CFU/mL) bacterial strains. Additionally, coated surfaces induced a significant inhibition of biofilm formation, an effect observed mainly for *E. coli*. Moreover, the pMPC coatings improved the capacity of PDMS to reduce the adhesion and proliferation of human macrophages by 50% and of human fibroblast by 40% compared to unmodified scaffold, circumventing undesired cell responses such as inflammation and fibrosis. All these highlighted the potential for the new PDMS–pMPC interfaces obtained by MAPLE to be used in the biomedical field to design new PDMS-based implants exhibiting long-term hydrophilic profile stability and better mitigating foreign body response and microbial infection.

## 1. Introduction

One of the most common strategies for surface modification of any medical device, in order to prevent undesired biological responses, involves using polymers-based coatings [1,2,3]. The applications related to antifouling surfaces or tailored cell adhesion include zwitterionic surface modification [2,4,5]. Among the zwitterionic polymers, the highly hydrophilic and low toxic 2-methacryloyloxyethyl phosphorylcholine (MPC) polymer has been used for intravascular stents, artificial hearts, contact lenses, oxygenators, breast implants, and hip acetabular liners [5,6,7,8,9,10,11,12,13,14,15,16]. Besides its hydrophilicity, MPC can provide electrically neutral surfaces with great potential to exhibit long-term anti-biofouling properties-suppressed protein, cells and bacterial adhesion, due to the hydration layer formed on the coated surface as a result of the phosphorylcholine groups present in MPC structure [8,9,10,11,12]. Moreover, a significant advantage is given by the ability of phosphorylcholine to induce bulk-like behaviour in the surrounding liquid environment and also to enhance the lubricant property of the material, improving its performance [12]. Some reports in recent years demonstrated that pMPC based surfaces, including grafting polymer layer (i.e., polymer brush structure, extending normal to the underlying substrate), provide new surface properties such as enhanced wettability, lubricity, or reduced biological responses. Being suitable for polymerisation or copolymerisation, MPC can be tailored to obtain coatings with antifouling properties. Various physical or chemical methods have been used for MPC grafting, from photo-induced radical polymerisation, thermal-induced radical polymerisation, dip-coat, spin-coat and plasma technology to salinisation. Most of them involve grafting of MPC or its copolymers on the surface, resulting in multiple thicknesses and hydrogel density [16], copolymerising 2-methacryloyloxyethyl phosphorylcholine (MPC) and dimethylsiloxane (DMS) units [17]. Other examples include biodegradable zwitterionic poly(2-methacryloyloxyethyl phosphorylcholine) (PMPC) nanogels with a thickness of 200 nm fabricated by the reflux precipitation polymerisation, aiming at drug delivery applications with excellent protein adsorption resistance, redox-responsive performance and stability under specific conditions [18]. Another approach was to use multistep surface functionalisation by firstly stabilising the surface with epoxide groups by modifying the Polydimethylsiloxane (PDMS) with a high-Mw poly(glycidyl methacrylate) linker. The second step involved using an ATRP initiator bearing a hydroxyl group attached to the surface in order to introduce alkyl bromide groups to initiate the polymerisation of vinyl monomers. Finally, zwitterionic MPC hydrogels could be grafted via SI-ATRP [19].

Polydimethylsiloxane (PDMS) is currently used as a silicon-based implant and in different medical devices due to its optical and mechanical properties, biocompatibility and resistance to biodegradation. However, its hydrophobic character is the main disadvantage of practical application. To prevent microbial infection from occurring on hydrophobic PDMS, hydrophilic material surface preparation is required. Using physical methods to immobilise pMPC on PDMS can be tricky and challenging. 

Previous reports showed that, by using MAPLE for physically immobilising a wide variety of compounds, surfaces coatings could be tailored in terms of thickness, roughness, and stability in an aqueous environment [20,21,22,23,24]. Even if exposure to UV or O_2_ plasma can lead to the transition toward a hydrophilic PDMS interface, the recovery of its hydrophobicity occurs within hours or days, accompanied by changes or even cracks on the surface [25]. 

The capacity of the optimised pMPC polymer layer deposited on PDMS surfaces to reduce bacterial adhesion and subsequent biofilm formation was tested for Gram-positive bacteria, *Staphylococcus aureus ATCC* 25923 (*S. aureus*), and Gram-negative bacteria, *Escherichia coli* ATCC 25922 (*E. coli*) strain. The assessment of adhesion, proliferation and morphology of cells grown on different surfaces was performed in vitro, using human THP-1 differentiated macrophages and human normal CCD-1070Sk fibroblasts (cells involved in foreign body reaction). 

In this context, in our study, we have investigated whether the pMPC polymeric layers obtained by MAPLE on the PDMS surface can lower the hydrophobic profile of the untreated PDMS surface, and its behaviour in time as compared to those deposited on the UV–ozone treated PDMS. 

## 2. Materials and Methods

### 2.1. Polymerisation of 2-Methacryloyloxyethyl Phosphorylcholine (MPC)

The 2-methacryloyloxyethyl phosphorylcholine (MPC) (Aldrich, St. Louis, MO, USA, 97%) was purified by recrystallisation from acetonitrile [26]. The initiator of the polymerisation reaction, potassium persulfate (K_2_S_2_O_8_) (KPS) (Aldrich), was recrystallised from ethanol and then vacuum-dried until constant mass. Typically, 0.1 g of MPC was dissolved in 5 mL of deionised water to which 0.005 g of KPS was added. After the complete solubilisation of the monomer and initiator, the mixture was nitrogen purged and then maintained for 6 h under continuous stirring at 80 °C. To separate poly(2-methacryloyloxyethyl phosphorylcholine) (pMPC), the viscous solution was precipitated in 5 mL absolute ethanol. The gel-like polymer was separated by centrifugation and vacuum dried until constant mass.

### 2.2. pMPC Coatings Obtained by MAPLE Method

**Figure 1:**
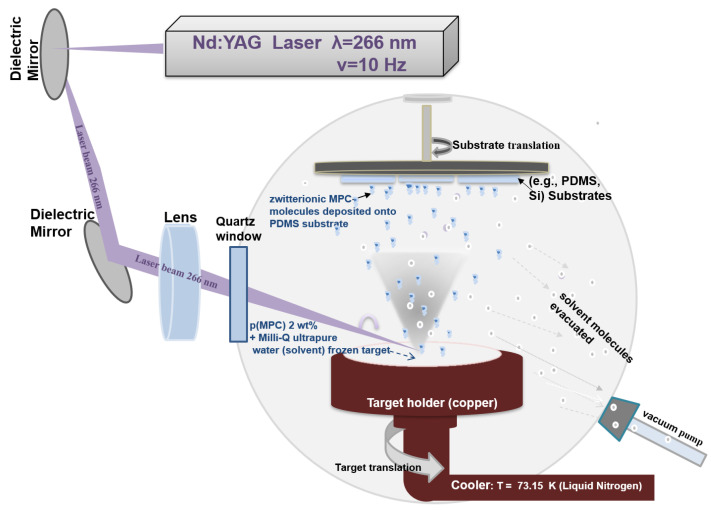
The experimental MAPLE set-up for obtaining pMPC coatings.

In this work, the MAPLE set-up consisted of a Nd:YAG pulsed laser system (Continuum SURELITE II™) (266 nm wavelength, 10 Hz repetition rate, 6 ns pulse duration) and a vacuum deposition chamber in which the typical target was introduced and maintained frozen (Figure 1). The PDMS substrates were placed at a distance of 3 cm and in parallel with the target surface. For coatings deposition, the laser beam scanned the cryogenic target surface through a lens with a focal length of 75 mm. 

The target consisted of pMPC polymer dissolved (2 wt.%) in Milli-Q ultrapure water, and frozen by liquid nitrogen in a copper target support. The target was maintained frozen during the deposition time by using liquid nitrogen and a cooling system. Vacuum pumps are used for removing the volatile molecules of the water. The laser spot measured at the target level was maintain at 1 mm^2^, and the used fluences were 250, 350, 450 and 650 mJ/cm^2^. The number of pulses was maintained at 72 k pulses, resulting in coatings with mediated thicknesses of 190, 270 and 346 nm (for 350, 450 and 650 mJ/cm^2^) as determined by AFM. The thickness of the coatings obtained at 250 mJ/cm^2^ could not be estimated as the coatings were not continuous.

### 2.3. PDMS Flat Substrates Preparation and UV–Ozone Treatment

Polydimethylsiloxane (PDMS; Sylgard 184 Silicone Elastomer Kit; Dow Corning, USA MI; 1:10) was placed in a Petri dish for 48 h at room temperature, and then heated at 80 °C for 1 h. The resulting PDMS samples were subsequently removed, cut into 1 × 1 cm^2^ pieces and cleaned by ultrasonication, twice, with each 10 min cleaning step being performed in Milli-Q ultrapure water.

The PDMS substrates obtained were cut into 1 cm^2^ samples. Sets of untreated PDMS samples, and several sets subjected to 2 h UV–ozone lamp cleaner (model UVC 104, NanoBioAnalytics Bürgel, Saale-Holzland, Germany), were used for MAPLE experiments.

### 2.4. pMPC Morphological Analysis

#### 2.4.1. Scanning Electron Microscopy (SEM)

The pMPC coatings on Si and PDMS were air-dried and covered using a sputtering coater with 10 nm Au (Agar Scientific Ltd., Essex, UK) prior to SEM measurements. Top view investigations of the pMPC surfaces were carried out by means of (SEM) with a JSM-531 Inspect S. System (Hillsboro, OR, USA) at an accelerating voltage of 20 kV. 

#### 2.4.2. Atomic Force Microscopy (AFM) 

AFM (XE 100, Park Systems, Suwon, South Korea) measurements were used for analysing the surface morphology and the overall roughness (quantified by the root mean square roughness) of the pMPC coatings. Imaging was performed in non-contact mode, using silicon tips, in ambient conditions. 

### 2.5. Wettability Characterisation by Contact Angle (CA) and Surface Energy Measurements

The wetting behaviour of the PDMS samples, with and without UV–ozone treatment, with and without pMPC coatings, was achieved by surface contact angle (CA) measurements by using the classic sessile drop method applied in ambient conditions. CA measurements were conducted using a KSV CAM101 microscope (KSV Instruments Ltd., Espoo, Finland) system equipped with a video camera and FireWire interface (resolution of 640 × 480 pixels). Milli-Q ultrapure water and di-iodomethane (Sigma Aldrich, St. Louis, MO, USA) were used for measuring the CA in order to determine the surface free energy (SFE) by the Owens, Wendt, Rabel and Kaelble (OWRK) method [27,28,29].

### 2.6. Chemical Profile: Analysis of pMPC

#### 2.6.1. Fourier Transform Infrared Spectroscopy (FTIR) Analysis

The specific chemical structure of the pMPC coatings obtained by MAPLE was investigated by the Fourier Transform Infrared Spectroscopy (FTIR) method using a JASCO (Jasco FTIR 6300/ Type A Spectrometer). Drop casted material was used as a reference for determining the main characteristic IR vibrations of functional groups of the pMPC. All the samples were measured in the 600–4000 cm^−1^ range, with a resolution of 4 cm^−1^, in transmission mode, while those for drop-cast were measured in ATR mode (Attenuated Total Reflectance).

#### 2.6.2. X-ray Photoelectron Spectroscopy (XPS) Analysis

To study the surface chemistry of layers, an Escalab Xi+ system (Thermo Scientific, Waltham, MA, USA) was used for X-ray photoelectron spectroscopy (XPS) survey and high-resolution XPS spectra acquisition. All the survey scans were acquired using an Al Kα gun with a spot size of 900 µm, and a pass energy of 50.0 eV with an energy step size of 1.00 eV (5 scans). The excessive charging load of the layers was reduced with the help of a flood gun. In the case of the high-resolution XPS spectra data, the pass energy used was set to 10.0 eV and the energy step size was 0.10 eV with 15 scans that were accumulated for O1s, N1s and C1s.

### 2.7. Biological Investigations 

#### 2.7.1. Antimicrobial Assay

The antimicrobial effect of pMPC was determined by standard cultures of Gram-positive bacteria, *Staphylococcus aureus* (*S. aureus*) ATCC 25923, and Gram-negative bacteria, *Escherichia coli* ATCC 25922 (*E. coli*). The bacterial suspensions, 108 CFU/mL, were prepared in PBS (Phosphate-Buffered Saline). From each suspension, 1 mL/sample was aspirated and placed over the 3 types of aliquots: positive control (free bacterial suspension), PDMS and PDMS–pMPC and were incubated for 24 h and 72 h at 37 °C. Following that, samples with attached microorganisms were extracted and vortexed until the microorganisms were detached from the treated surfaces. Serial dilutions of the samples were plated onto a Plate Count Agar and incubated for 24 h and 72 h at 37 °C. By counting the number of colonies from the plate, the number of bacteria in the bacterial suspensions was calculated, also considering the dilution factor. 

#### 2.7.2. Antibiofilm Effect

The antibiofilm effect of PDMS–pMPC was observed by using SEM. Briefly, the samples were fixed in ethanol dilutions (35–100%) to visualise biofilm formation after 24 h and 72 h of incubation at 37 °C in the presence of the two standard microbial suspensions: *S. aureus* and *E. coli*.

### 2.8. Cell Culture Models

THP-1 cells (ATCC, TIB-202 cell line), human monocyte cells derived from a patient with acute monocytic leukaemia, were maintained in RPMI 1640 medium supplemented with 10% (*v*/*v*) inactivated foetal bovine serum (FBS) and 1% (*v*/*v*) penicillin-streptomycin (10,000 Units/mL–10,000 μg/mL) (all Gibco by Life Technologies, Thermo Fisher Scientific, Waltham, MA, USA) at 37 °C in a humidified atmosphere of 5% CO_2_ as described previously [30]. THP-1 cells (4 × 105 cells/surface material) were differentiated to macrophages by incubation with 100 ng/mL phorbol 12-myristate-13-acetate (PMA, Sigma-Aldrich, Saint Louis, MO, USA, P8139) for 48 h in 24-well tissue culture plates (Costar, Corning Inc., Corning, NY, USA), gently washed with cell medium and left to rest for 4 h in glutamine-free RPMI medium supplemented with 5% (*v*/*v*) FBS. Then, cells were activated by exposure to 50 ng/mL lipopolysaccharide (LPS, *Escherichia coli* 055:B5, Sigma L4524, Sigma-Aldrich, Saint Louis, MO, USA) for a further 18 h to simulate pro-inflammatory experimental conditions. 

Human normal fibroblast CCD1070-Sk cells were cultured in α-MEM medium supplemented with 10% (*v*/*v*) FBS and 1% (*v*/*v*) streptomycin/penicillin (all from Gibco Life Technologies, NY, USA) and kept at 37 °C with 5% CO_2_. The medium was changed twice a week. Cell viability was monitored using 0.4% trypan blue dye staining and cells with more than 90% viability were used for this study.

#### 2.8.1. Assessment of Cell Metabolic Activity 

Prior to the biological experiments, all materials were sterilised by immersion in 1% penicillin-streptomycin solution for 15 min in order to prevent microbial contamination.

CellTiter 96^®^ Aqueous One Solution Cell Proliferation Assay-MTS (Promega, Fitchburg, WI, USA) was used for the evaluation of cell metabolic activity following the manufacturer’s instruction. MTS is a colorimetric method for sensitive quantification of viable cells based on tetrazolium compound (3-(4,5-dimethylthiazol-2-yl)-5-(3-carboxymethoxyphenyl)-2-(4-sulfophenyl)-2H-tetrazolium) reduction to formazan by dehydrogenases present in the metabolically active cells. The amount of formazan released into the culture medium is proportional to the number of live cells. Macrophage-differentiated THP-1 cells (4 × 10^5^ on each material), treated or not with LPS and CCD-1070Sk cells (5 × 104 /surface material), were gently washed with medium and then incubated with MTS solution at 37 °C. After 30 min, 100 μL of supernatant was transferred to a 96-well plate, and the optical density was measured at 450 nm using a microplate reader (Mithras Berthold LB 940, Berthold Technologies, Bad Wildbad, Germany).

Possible cytotoxic effects of scaffolds on studied cell lines were evaluated also by indirect contact. PDMS and PDMS–pMPC materials were individually placed in a 24-well plate in cell culture media for 24 h at 37 °C. Previously, THP-1 cells differentiated to macrophages for 48 h and CCD1070SK cells seeded for 24 h before the experiment were incubated for 1 day and 3 days, respectively, with supernatants obtained from direct contact of scaffolds with cell culture media. Afterwards, MTS quantitative assay was performed on confluent cells to evaluate metabolic active cells as described before. Cell viability was expressed as percent of control (cells grown on the glass coverslip and incubated with cell media).

#### 2.8.2. Evaluation of Cell Adhesion and Morphology

The adhesion and morphology of THP-1 differentiated macrophages and CCD-1070Sk fibroblasts cultured on PDMS scaffolds (with or without pMPC) and control samples (coverslip) were investigated by fluorescence microscopy. Cells adhered on all samples were washed with PBS, fixed with 4% paraformaldehyde at room temperature for 15 min, permeabilised with 0.2% TritonX-100 for 3 min and blocked in 0.5% bovine serum albumin (BSA) in PBS. Alexa Fluor 488-conjugated Phalloidin (1:50, A 12379 Invitrogen, Thermo Fisher Sci., CA, USA) staining was used for actin filaments labelling (green), while for nuclei staining (blue), cells were treated with 1 μg/mL Hoechst (H 21492 Life Technologies, Molecular Probes, Eugene, OR, USA). After each step of incubation, cells seeded on materials were rinsed with PBS. Before image acquisition, the specimens were mounted in ProlongGold Antifade Reagent (P 36934 Molecular Probes, Life Technologies, Eugene, OR, USA). Fluorescence images were acquired using a Zeiss Axiocam ERc5s Apotom microscope with a 40× objective. The images were analysed with the AxioVision Rel. 4.8 software (Zeiss, Jena, Germany).

For indirect contact experiments, THP-1-derived macrophages and CCD1070SK fibroblasts exposed to extracts obtained by direct incubation of scaffold materials with media were subjected to light microscopy investigation. For cell morphology, the whole area of the sample was scanned with a Zeiss AxioImager Z1 inverted microscope controlled by Tissue FAXS iPlus system (TissueGnostics GmbH, Vienna, Austria). Relevant micrographs acquired at 10× magnification (scale bar = 100 µm) were presented.

For SEM analysis, THP-1 differentiated macrophages and CCD-1070Sk fibroblasts cultured on CTRL, PDMS and PDMS–pMPC materials were washed with PBS and fixed with 2.5% glutaraldehyde solution for 20 min. The samples were gradually dehydrated with 70%, 90%, and 100% ethanol solutions for 15 min, twice for each concentration followed by two rounds of 3 min incubation with 50%, 75% and 100% hexamethyldisilazane (HDMS, Sigma-Aldrich, St. Louis, MO, USA) solution in ethanol. All steps and evaporation of the HDMS solution were carried out in a Euroclone AURA 2000 M.C. hood (Euroclone SpA, Siziano, Italy). Images were acquired using an Inspect S Electron Scanning Microscope (FEI Company, Hillsboro, OR, USA).

### 2.9. Statistical Analysis

Statistical analysis was performed with GraphPad Prism 4 software (GraphPad, San Diego, CA, USA) using one-way ANOVA with Bonferroni’s multiple comparison tests. Triplicate samples were used in all biological assays. The data are presented as means + SD (standard deviation). The *p* values < 0.05 were considered to be statistically significant.

## 3. Results

### 3.1. Morphological Characterisation of pMPC Coatings Obtained by MAPLE on Si and PDMS

**Figure 2:**
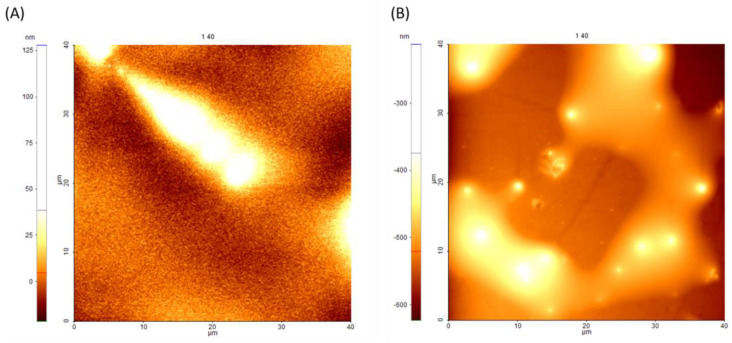
Atomic force microscopy images of pMPC dropcasted onto (**A**) Si with smooth, homogenous and random linear microfeatures surfaces; and (**B**) onto Si PDMS surface, characterised by the accumulation of pMPC microislands onto the surface. Scan area: 40 × 40 μm^2^.

The interfaces of Si–pMPC and PDMS–pMPC obtained by the casting of the polymer are different accordingly to the substrate used. Thus, while the typical interface of Si-pMPC is relatively flat, with a mainly smooth surface (average surface roughness (Ra) below 10 nm for 40 × 40 μm) but with some linear characteristics (Figure 2A), or island-like material accumulation in an aleatory manner on PDMS, material accumulates in large-sized islands (Figure 2B). The layer outcome smooth topography of casted material on Si is similar to the one reported by Dunn et al. [31] which refers in general to a gel layer with a near 100% water content on the surface, due to highly hydrated gel surfaces, while the hydrophobicity of the PDMS substrates leads to the accumulation of material. 

**Figure 3:**
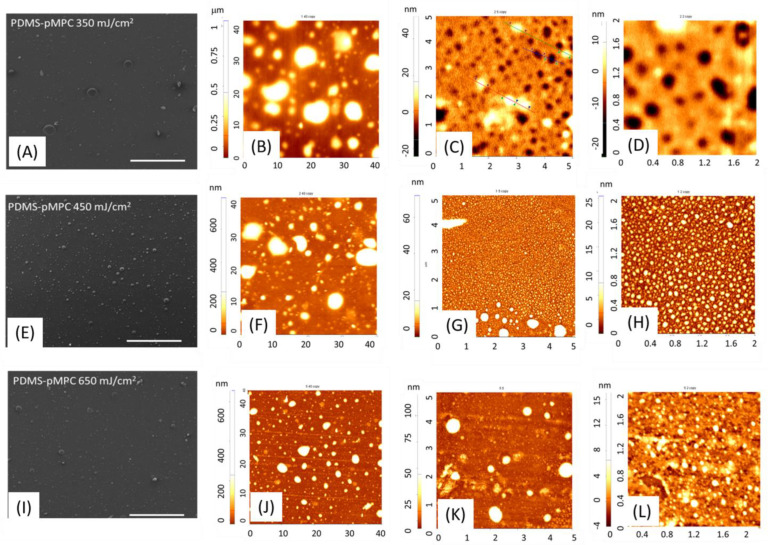
SEM and AFM images of pMPC coatings obtained by MAPLE on untreated PDMS, using fluences of 350 mJ/cm^2^ (**A**–**D**), 450 mJ/cm^2^ (**E**–**H**) and 650 mJ/cm^2^ (**I**–**L**). SEM magnification 1000×, scale bar SEM images 300 μm. Scan areas for AFM: 40 × 40 μm^2^, 5 × 5 μm^2^ and 2 × 2 μm^2^ for the pMPC coatings obtained at fluences of 350 mJ/cm^2^ (**B**–**D**), 450 mJ/cm^2^ (**F**–**H**) and 650 mJ/cm^2^ (**J**–**L**).

**Figure 4:**
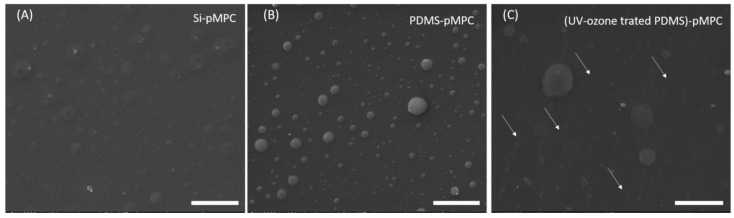
SEM images of pMPC coatings obtained by MAPLE at 450 mJ/cm2 onto Si (**A**), PDMS (**B**) and UV–Ozone treated PDMS (2 h) (**C**). The white arrows indicate the wave-like structures observed for the coatings deposited on UV–Ozone treated PDMS. Scale bar: 10 μm.

Nevertheless, SEM on a large area, as well as AFM (40 × 40 μm^2^) (Figure 3) images, revealed that pMPC coatings obtained by MAPLE exhibited different microfeature surfaces depending both on the laser fluence (Figure 3) and substrate (Figure 4). The new interfaces obtained by MAPLE exhibited distinctive surface features and topographies resulting from densely packed MPC polymer units attached to the PDMS surface, with interspersed micro and nanoglobular domain features, exhibiting roughnesses in the range of 55–184 nm, except for the coatings obtained with 250 mJ/cm^2^ (Figure S1), which were characterised by discontinuous surfaces, with the island-like material accumulation. Additionally, the distinctive trend observed for the coatings obtained by MAPLE by varying the fluence is surface features with interspersed micro and nanoglobular domain features, characterised by a decrease in globular structures presence with increasing fluence; respectively, a decrease of the root mean square roughness (Rq) from 184 nm for 350 mJ/cm^2^ and 119 nm for 450 mJ/cm^2^ to 55 nm for 650 mJ/cm^2^ (Figure 3). 

However, when 2 × 2 μm^2^ areas are considered, differences between surface features are shown, from nanopores (Figure 3C,D) to randomly distributed nanoglobular features (Figure 3F,J), as well as to an assembling of droplet patterns (Figure 3G,H). An explanation could be given by the fact that, due to the zwitterionic nature of MPC, condensation as micro-scaled liquid droplets take place on the hydrophobic surface of PDMS [32]. In general, for MAPLE deposition, the typical tendency is an increase in average surface roughness with increasing fluence, but in the present work, the increase of the fluence led to the roughness decrease. The observed trend in the case of pMPC can be explained by the zwitterionic nature of the material. Moreover, as the accumulation of nanoglobular microfeatures surfaces and thickness increases with fluence value, the hydration tendency given by its hygroscopic nature is favoured due to the material density on the surface. The differences observed for the coatings obtained at 250 mJ/cm^2^ can be explained by the fact that the surface tension of PDMS substrate cannot be exceeded by the thin layer of the pMPC material deposited, and the final coating appears as reorganised discontinuous islands with 190–200 nm thickness on PDMS (Figure S1). As previously mentioned, the surface energy of a substrate might also influence the coating morphology, together with the morphology characteristics of the treated substrate. This is particularly interesting in the PDMS case, as its surface characteristics, from hydrophobicity recovery to surface micro and nanofeatures, are linked to the surface treatment time and method. In our case, the coatings obtained by MAPLE at 450 mJ/cm^2^ on Si and UV–ozone treated PDMS are characterised by a smooth surface, with increased globular features observed mainly for untreated PDMS. However, when UV–Ozone treated substrates were used, the observed wave-like features (Figure 4 and Figure S2) are attributed to the specific modifications induced by the surface treatment. It is known that, due to plasma or UV–ozone treatment, the PDMS surface oxidises, forming a thin, stiff silicate layer on the surface [33]. As PDMS cools, contraction occurs, inducing compressive stress, and, respectively, relieving. This final step induces a buckling, and therefore wave-like features were observed (marked by the white arrows), as reported in the literature [33,34].

### 3.2. FTIR and XPS of pMPC Coatings Evaluation

**Figure 5:**
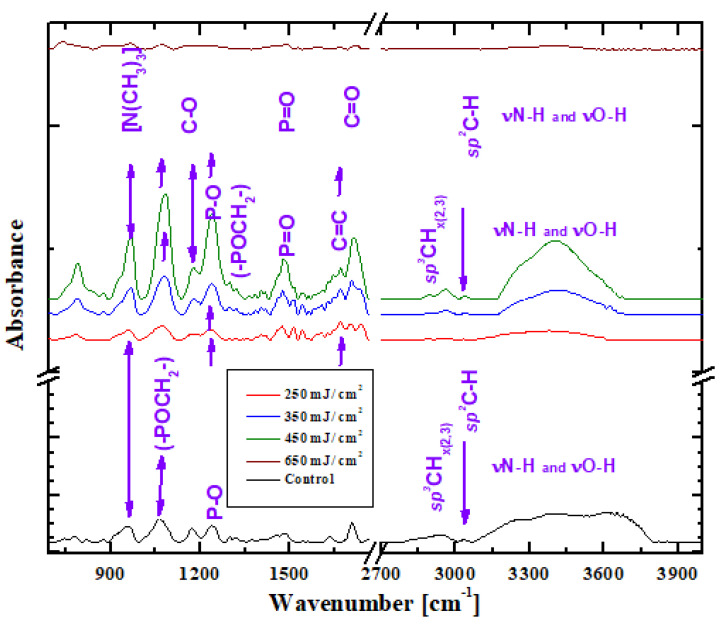
FTIR spectra of pMPC coatings obtained by MAPLE on Si as compared to the initial material.

The infrared spectra of the typical vibrational bands of the MPC polymer can be visualised in Figure 5. The MPC films obtained by the laser technique not only keep the chemical bonds of the initial material but, according to the FTIR spectra, have prolonged chemical stability in the ambient atmosphere. Moreover, it can be observed that, using a higher fluence of the laser (450 mJ/cm^2^), a replica can be obtained that is almost chemically identical to the initial material. The FTIR spectrum of the initial material (black line) shows absorptions for the trimethylammonium group, respectively, in the phosphorylcholine group at 958 cm^−1^ [N(CH_3_)_3_], 1079 cm^−1^ and 1298 cm^−1^ (-POCH_2_-), 1174 cm^−1^ C-O and 1239 cm^−1^ for the P-O-C deformation vibration [35,36]. Moreover, the initial material shows the vibrations of the bonds P=O la 1482 cm^−1^, C=C la 1636 cm^−1,^ and C=O at 1728 cm^−1^ [35]. Between 2850 and 3000 cm^−1^, sp^3^ CH_x(2,3)_ types occur, and at 3038 cm^−1^ there is an sp^2^ vibration bond type, C-H. Additionally, the vibrations of the νN-H and νO-H occur between 3080 and 3700 cm^−1^. The maintenance of the functional groups at the surface of the polymer membranes was confirmed by the spectra of the deposited coatings by MAPLE. The spectra of the pMPC surfaces of MAPLE obtained coatings showed a phosphorylcholine group of the MPC unit, with the absorption lines at 958 cm^−1^ corresponding to C-N stretching vibration (-N+ (CH_3_)_3_), 1079 cm^−1^, and 1298 cm^−1^ (-POCH_2_-). Additionally, from the MAPLE obtained samples, it can also be observed that the methacrylate structures of the polymer backbone are given by the carbonyl band at 1720 cm^−1^, while the PO unit can be found at 1239 cm^−1^.

**Figure 6:**
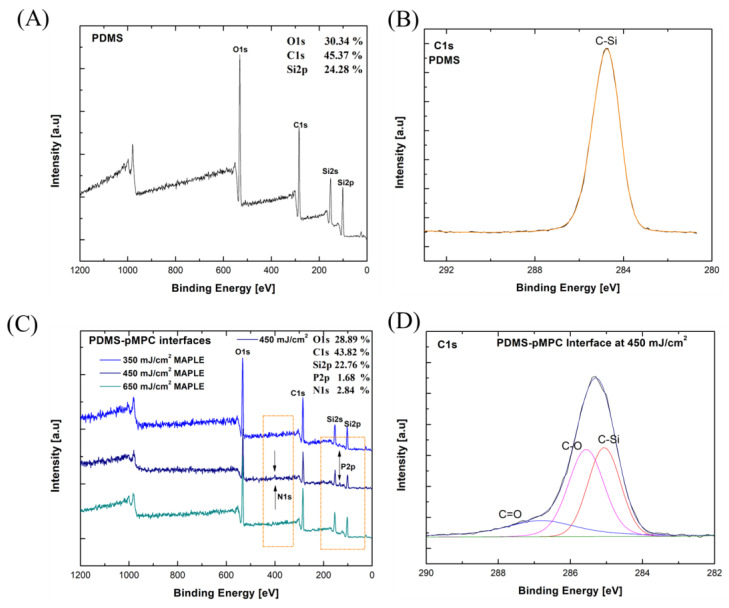
XPS analysis of PDMS interface: (**A**) XPS full spectra (black survey), (**B**) C1s high resolution spectra with (**C**) XPS full spectra (coloured survey) of PDMS–pMPC interfaces deposited by MAPLE method at different laser fluences, and (**D**) C1s spectra of PDMS–pMPC interface at 450 mJ/cm^2^.

**Figure 7:**
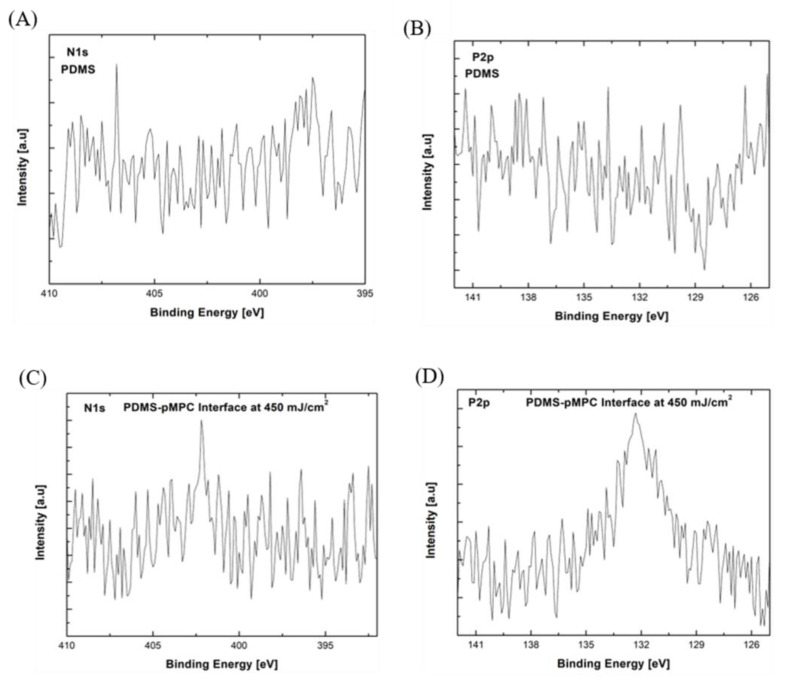
XPS high resolution spectra comparison of the PDMS interface; (**A**) N1s spectra, and (**B**) P2p spectra, and PDMS–pMPC interfaces deposited by MAPLE at 450 mJ/cm^2^; (**C**) N1s spectra, (**D**) P2p spectra.

XPS evaluation of pMPC coatings on PDMS (Figure 6) and Si (Figure 7) substrates reveals that the full spectra (Figure 6A) of uncoated PDMS substrate primarily contained three representative signals; that is, carbon (C1s) at 284.8 eV, oxygen (O1s) at 530 eV, and silicon (Si) at 103 eV. As compared with the pure PDMS interface, the corresponding peaks of nitrogen (N1s) and phosphorus (P2p) orbital elements of the hydrophilic phosphorylcholine groups [37,38] were observed at the PDMS–pMPC interface in the case of the coatings obtained by the MAPLE technique (Figure 6B). The survey scans of the PDMS–pMPC substrates obtained by MAPLE revealed an increase in carbon (C1s) and oxygen (O1s) elemental composition and a corresponding decrease in silicon (Si), as well as the corresponding peaks of nitrogen (N1s) and phosphorus (P2p) elements of the hydrophilic phosphorylcholine groups, especially in the case of the coatings obtained for a fluence of 450 mJ/cm^2^. Nevertheless, the uncoated PDMS spectra featuring only C1s peaked at 284.8 eV, corresponding to C-Si bonds (Figure 6B). In contrast, PDMS–pMPC at the 450 mJ/cm^2^ laser fluence showed both a strong peak near 284.9 eV arising from C-Si bonds and a shoulder near 286.3 eV, corresponding to C-O bonds, where the intensity of the C-O (285.3eV) peak increased, and a new peak corresponding to C=O (287.3 eV) appeared. The existence of all these peaks indicates the existence of MPC segments from the PDMS–pMPC presence near the surface [39]. The elemental ratios of N1s and P2p element composition of PDMS–pMPC coating (450 mJ/cm^2^) were around 2.84% and 1.68%, where the representative peaks of N1s and P2p were from XPS spectra (Figure 6C). The experimental N/P ratio is similar to the reported theoretical value of 1.0 [40].

Additionally, for the pMPC-Si interfaces obtained at the fluence of 450 mJ/cm^2^, where the representative peaks of N1s and P2p from the XPS spectra (Figure S3) were observed, the elemental ratios of N1s and P2p are 2.37% and 5.28%, respectively.

The high-resolution C1s peaks observed at 286.2 eV and 287.9 eV are assigned to C-O and C=O bonds, respectively, for the coatings obtained by the MAPLE process (Figure 6D and Figure 7D).

Besides the common elements found in both substrate and pMPC polymer coating, an additional peak of the N1s spectra was found at 402.4 eV, which corresponds to the functional group of N^+^(CH_3_)_3_ [39] (PDMS–pMPC Figure 7A,C and pMPC-Si Figure S4A,C). Both the XPS and IR spectra indicated in mutual agreement that pMPC were deposited onto the PDMS surface. 

These results corroborated the FTIR analysis (Figure 5), and with morphological analysis indicate that the optimum pMPC polymer interfaces are obtained by MAPLE deposition for the laser fluence of 450 mJ/cm^2^.

### 3.3. Wettability 

pMPC is known as a biocompatible polymer with an amorphous nature and super hydrophilicity characteristics. However, in our case, the influence of the substrate’s characteristics on the final wettability of the PDMS–pMPC, even if the polymer is known to induce superhydrophilicity characteristics, leads to moderate hydrophilicity of the PDMS coated surfaces, except the coatings obtained for 350 mJ/cm^2^, where a value of 96 degrees was measured for the contact angle. Nevertheless, the reduction in the hydrophobic profile of the surfaces given by the coatings obtained by MAPLE is up to 40% on untreated PDMS surfaces. In comparison, for treated PDMS the percentage goes up to 60%. On Si, a contact angle as low as 17 degrees is obtained (Supplementary Materials, Figure S5), indicating a reduction of more than 70%, confirming that the increased hydrophilicity of the films is attributed to the ionic hydration of the phosphorylcholine group in the MPC unit. 

UV–ozone treated surfaces were used only as controls to demonstrate the importance of the underlying substrates’ wettability, and were not considered for biological evaluation, given the known fact of hydrophobicity recovery of PDMS surfaces, as presented in the Supplementary Materials (Figures S6 and S7). Nevertheless, UV–ozone treatment can create structures of cracks on the PDMS surface, as a result of the migration of low molar mass siloxanes to the surface, as well as due to an overlap of various nano-mechanical, and diffusional, processes [41,42].

The change in surface wettability characteristics of PDMS after pMPC coatings is based on the known ability of PC groups to induce bulk-like behaviour in the surrounding water. Therefore, depending on the coatings’ characteristics, we assume that dense coatings allowing better hydration will allow the molecules within the bulk of the liquid to affect the neighbouring molecules in all directions so that there is no net force on the molecule. In contrast, a molecule at the surface will experience only net inward forces [41]. 

**Figure 8:**
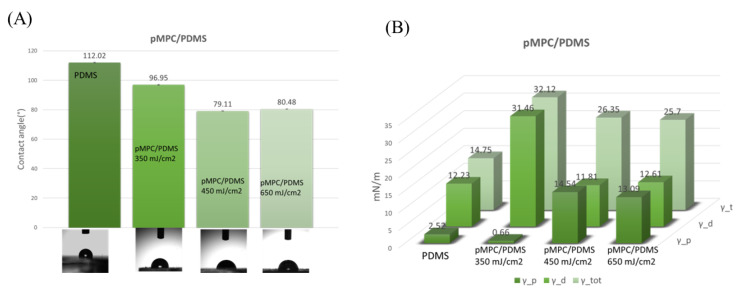
Histograms of contact angle measurements onto PDMS and pMPC coatings onto untreated PDMS (**A**), and the corresponding surface energy (**B**).

It is a known fact that the cellular interaction at an interface is affected by the surface energy (i.e., cell adhesion and elongation), the energy needed for recruiting new adhesive molecules, and the elastic energy, which opposes adhesion and elongation of the cell membrane. Therefore, the cell spread length on a surface can be determined by modifying the total free energy of the cell–substrate system. Using the OWRK method [27,28], surface free energies (SFE) as well as the polar and dispersive components of the SFE were determined for the tested materials. It was found that pMPC layers obtained at a fluence higher than or equal to 450 mJ/cm^2^ presented the largest polar component (Figure 8B). On UV ozone treated PDMS, followed by coating with pMPC, no significant differences were obtained for the polar component (Figure S6). However, the hydrophobicity of the PDMS substrate influenced the top pMPC layer, leading to an increase in the contact angle by more than 30%, all the surfaces becoming hydrophobic (Figure S7). SEM images of pMPC coatings deposited on PDMS after immersion in water for 72 h showed that, although the surface suffered modifications, mostly being characterised by the disappearance of the microglobular structures, the coatings were not completely removed from the PDMS surface (Figure S8).

Based on the result of high-resolution scans, XPS measured on the uppermost surface, elemental composition, contact angle and degradation of pMPC coatings evaluation presented in the Supplementary Information (Figures S6–S8), the best results were obtained for the pMPC deposited by MAPLE at the laser fluence of 450 mJ/cm^2^ on the PDMS and Si substrates. 

### 3.4. In Vitro Biological Behaviour

#### 3.4.1. Microbial-Material Surface Interaction

The adhesion of bacteria, and proliferation over time on implant surface, could become a problem, causing its failure. Bacterial adhesion is a complex process dependent on implant surface properties such as charge, hydrophobicity/hydrophilicity, and topography [43,44,45,46,47,48]. Materials covered with neutral zwitterionic MPC polymer provide a super hydrophilic surface through the formation of a hydration layer able to avoid biofouling adhesion. To prevent bacterial infection, it is important to inhibit their initial adherence on the implant surface. Here, we have investigated the influence of PDMS and pMPC-coated PDMS on microbial adhesion using *E. coli* and *S. aureus* as a model for Gram-negative and Gram-positive bacteria, respectively. The adherence of microorganisms to scaffolds was evaluated by the bacterial colony-counting method (Colony Forming Units, CFU) after 24 and 72h of incubation. 

**Figure 9:**
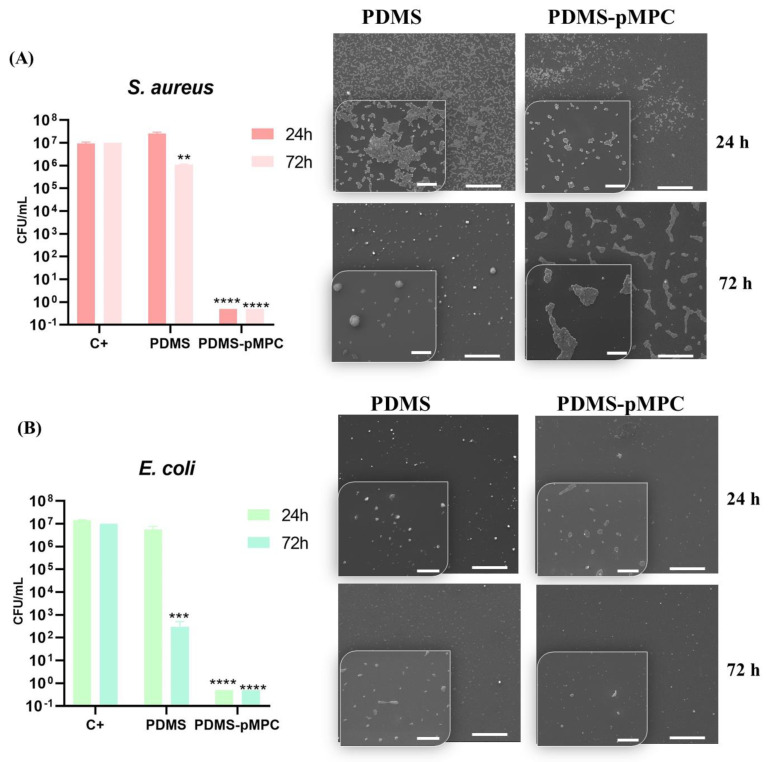
Colony forming unit (CFU) analysis of (**A**) *S. aureus* and (**B**) *E. coli* after 24 and 72 h treatment with PDMS loaded with MPC and soft PDMS. The positive control is represented by pure bacterial culture in the absence of substrates. SEM images are after 24 and 72 h of incubation with *S. aureus* and *E. coli*. Scale bar: 50µm (insert: 10 µm). Data expressed as mean ± SD, *n* = 3, (** *p* < 0.01, *** *p* < 0.001, and **** *p* < 0.0001 vs. C+).

As shown in Figure 9A,B a large number of bacteria attached to the Control (C+), with a significant decrease (**** *p* < 0.0001 vs. C+) in bacterial colonisation on MPC-functionalised PDMS surfaces, was observed for both bacterial strains investigated. Thus, compared to the control, the number of colonies after 72 h was decreased from 56 × 10^6^ CFU/mL to 2 × 10^3^ CFU/mL for *E. coli* and from 29 × 10^6^ to 3 × 10^2^ CFU/mL in the case of *S. aureus*. Uncoated PDMS was less efficient, the anti-adhesion effect being obtained only after 72 h, and this was more significant for *E. coli* (i.e., from 5 × 10^6^ CFU/mL at 24 h to 4.5 × 10^3^ CFU/mL at 72 h (*** *p* < 0.001)). However, in the case of *S. aureus*, the CFU values decreased less from 2.4 × 10^7^ CFU/mL after 24 h 11 × 10^5^ CFU/mL at 72 h (** *p* < 0.01). These observations can be linked to the fact that, besides the hydrophobicity of the material surface, bacteria adhesion is also linked to the hydrophobicity of the bacterial cell wall, growth medium, bacteria age, and bacterial surface structure. The hydrophobic surface of untreated samples is one of the main factors affecting the adhesion of bacteria on the surface of PDMS, and the observed decrease in the CFU values with the increase of the incubation time of the bacteria on the untreated PDMS surfaces could be linked to the water diffusion in the PDMS after 72 h, as hydrophilic materials are described as more resistant to bacterial adhesion than hydrophobic materials. 

These observations on PDMS–pMPC samples are in concordance with Qin et al., in their study reporting excellent antifouling and antibacterial properties of MPC gel layer grafted on PDMS, with low attachment of *E. coli* after 2 h of incubation with bacterial suspension [19] and significantly reduction of bacterial adhesion up to 200 cells/mm^2^. 

Lin et al. also noticed a higher number of *E. coli* on the pristine PDMS substrate than on the MPC-coated PDMS substrate [39]. In our case, the developed PDMS substrate functionalised with moderately hydrophilic pMPC coatings significantly inhibit the adhesion of both bacterial strains, *E. coli* and *S. aureus*. These observations can be explained by the effect of the steric hindrance formed by pMPC layers onto the PDMS, allowing it to exhibit an antibacterial effect.

It is also well known that microbial communities can grow and attach on an implanted material, its colonisation resulting in encapsulation, resistance against immune response or antibiotics therapy [41]. Therefore, the effect of biofilm colonisation on the PDMS–pMPC samples was evaluated by SEM analysis.

As shown in the SEM images (Figure 9), many microbial colonies grew on uncoated surfaces, while PDMS–pMPC surfaces significantly reduced bacterial colonisation after 24 h of incubation. Prolonging the incubation time to 72 h led to the significant inhibition of biofilm formation on coated surfaces compared to unmodified PDMS, especially in the case of *E. coli*. Only a few aggregated bacterial colonies that did not form a bacterial biofilm could be observed for *S. aureus*. Kaneko et al. [42] reported a suppression of initial bacterial adhesion of methicillin-resistant *S. aureus* on pMPC coated suture, and a significant biofilm formation only on the noncoated suture. The different behaviours could be attributed to the repulsive effect between different charges, hydrophobic properties of the surface of bacteria and hydrophilicity of the tested biointerfaces given by pMPC coatings, as well as to the presence of a phospholipid polar group similar to the cell membrane structure, known to reduce protein adsorption and bacterial adhesion, thereby preventing related infections. 

#### 3.4.2. Cell-Material Surface Interplay

The interaction of mammalian cells with implant surface and the controlling of their functions are important focal points of the biomedical devices development domain. Cell behaviour on biomaterial is influenced by surface properties such as chemistry, charge, hydrophobicity/hydrophilicity, and roughness [48,49,50,51,52,53,54,55,56,57,58,59]. The non-specific cell adhesion to implant surface is one of the causes leading to adverse body reaction and, finally, to implanted devices’ failure. Therefore, engineering the surface characteristics in order to reduce cellular adhesion and activation is of great interest and represents a major challenge for advanced biomaterial implants [60,61].

In this study, the interaction between PDMS and PDMS covered with the hydrophilic pMPC with cells was investigated using human THP-1differentiated macrophages and human normal fibroblasts CCD1070Sk as in vitro experimental models. These two cell lines were chosen since macrophages and fibroblasts are involved in foreign body reaction (inflammation, cell recruitment, and fibrosis) and implant degradation processes.

**Figure 10:**
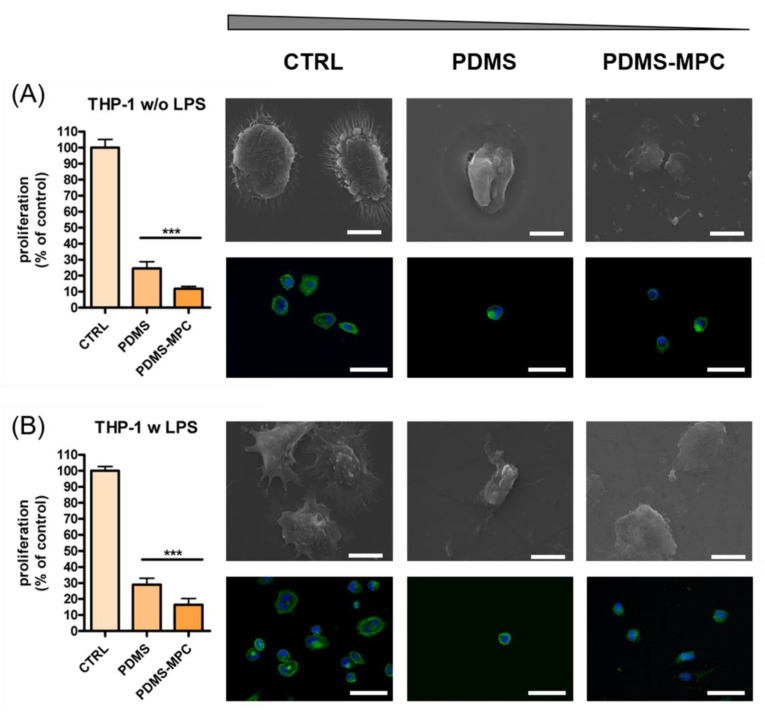
Proliferation, morphology and adhesion of THP-1 macrophages on PDMS scaffolds (without or with pMPC) in the absence (**A**) or presence of LPS (**B**). Cells cultured on glass coverslip served as control (CTRL). MTS data are shown as mean ± standard deviation values of triplicates in each group. *** *p* < 0.001. Representative fluorescence microscopy images of THP-1 cells cultured on all surfaces (objective 40×). Cells were labelled for actin (green) and nuclei (blue). Scale bar: 50μm. Representative SEM microscopy images of THP-1 cells’ morphology on surface materials were at 5000× magnification (Inspect S Electron Scanning Microscope). Scale bar: 10 μm.

The metabolic activity of cells grown on PDMS, PDMS–pMPC substrate and glass coverslip (CTRL) for 24 h was assessed by MTS assay. In the case of macrophages, the experiment was performed with cells untreated and treated with the pro-inflammatory stimulus, LPS, for 18 h. As seen in Figure 10A,B the proliferation of macrophages grown on PDMS and PDMS MPC was significantly reduced (over 80%) compared to CTRL, regardless of the presence or absence of LPS in the culture medium (*** *p* < 0.001 vs. CTRL). However, the reduction of proliferation was more obvious for the MPC coating with only 10–15% viable cells than 25–30% in the case of uncoated PDMS. 

Next, the influence of different biomaterials on THP-1 cells differentiated to macrophages adhesion and morphology was assessed by the fluorescence labelling of actin (green) filaments, a protein of the cytoskeleton involved in cell adhesion (Figure 10).

Numerous THP-1 differentiated macrophages in standard conditions (without LPS stimulation) presented largely round morphology with numerous thin filopodia filaments on CTRL substrate (coverslip) (Figure 10A). Contrary to that, reduced number of cells could be seen on PDMS and PDMS-MPC substrates, results that correlate with MTS data. Cells were predominantly rounded in morphology without actin filaments protruding from the cell periphery, but presented podosome-like structures suggesting adaptation of morphology to the substrate. No multinucleated cells were present on any investigated surfaces, indicating that none of the substrates induce foreign body response in standard conditions (Figure 10A).

In the case of LPS-stimulated THP-1 macrophages, numerous cells exhibited increased surface contact with CTRL substrate (Figure 10B). Macrophages presented enlarged surfaces compared to untreated cells (without LPS), mixed elongated or spherical morphologies with podosome structures and numerous long filopodia and lamellipodia protrusions. Binucleated cells can be found on CTRL support, suggesting a pro-inflammatory activation of the cells to a M1 phonotype. PDMS and pMPC-coated PDMS scaffolds induced in LPS-activated THP-1 cells a reduction of cell aria, small mononucleated cells presented round morphology with no expression of filopodia structures, suggesting a weak adherence to the substrate (Figure 10B). The morphological modification of both THP-1 differentiated macrophages, activated or not with LPS stimulus, observed by immuno-staining in the case of CTRL, PDMS and PDMS–pMPC scaffold samples, are similar to the one detected on SEM micrographs (Figure 10A,B).

An impaired attachment of the peripheral blood mononuclear cells’ differentiated macrophages induced by MPC functionalised PDMS was also reported by Qin et al. [19] A reduction in the macrophages’ attachment on the implant surface and the promotion of their polarisation toward an anti-inflammatory phenotype (M2) is needed to prevent foreign body reaction and to improve implant biocompatibility for the long term [50].

Similar behaviour was observed for CCD-1070Sk fibroblasts, but with a lesser effect on cell proliferation (* *p* < 0.05 vs. CTRL) than the one found for macrophages (Figure 10). However, unlike THP-1 cells, the proliferation of fibroblasts grown on PDMS surface covered with MPC was significantly reduced (over 45%) compared to the PDMS surface (• *p* < 0.05 vs. PDMS). Thus, the proliferation of cells grown on the PDMS surface was 75% for the control and 40% for MPC coating.

The results obtained in evaluating cell proliferation can probably be explained by a reduced number of cells present on PDMS-based materials, likely due to low adhesion forces and not to a cytotoxic effect. This is proved by the absence of cell cytotoxicity after incubation with PDMS and PDMS–pMPC extracts. Moreover, morphology and cells’ behaviour when treated with material extracts resembled the normal phenotype of each cell line investigated (Supplementary Figures S9 and S10). 

On such materials, cell-surface interactions are weak, and the cells can detach easily during the washing steps of the experimental protocol used. For both analysed cell lines the detachment was more evident in the case of pMPC-coated PDMS compared to unmodified PDMS, whose hydrophobic surface induces a higher level of interaction with cells than the hydrophilic MPC coating. A similar effect was reported by Qin et al. [19] in their study with human blood-derived macrophages cultured on PDMS functionalised with MPC.

**Figure 11:**
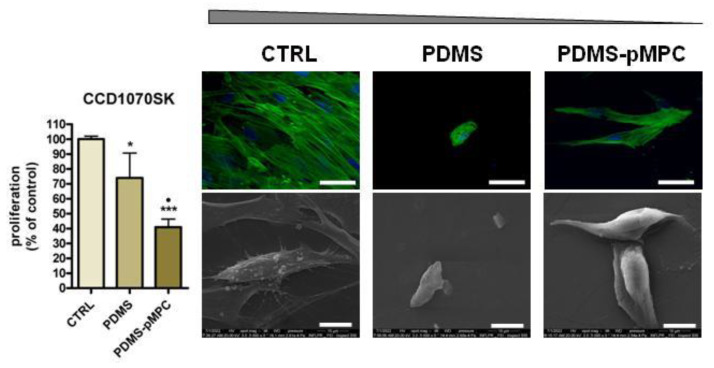
Proliferation, morphology and adhesion of CCD-1070Sk fibroblasts on PDMS scaffolds (with or without MPC). Cells cultured on glass coverslip served as control (CTRL). MTS data are shown as mean ± standard deviation values of triplicates in each group. *** *p* < 0.001, * *p* < 0.05 vs. CTRL, • *p* < 0.05 vs. PDMS surface. Representative fluorescence microscopy images of cells cultured on all surfaces (objective 40×). Cells were labelled for actin (green) and nuclei (blue). Scale bar 50 μm. Representative SEM microscopy images of fibroblasts’ morphology on surface materials at 5000× magnification (Inspect S Electron Scanning Microscope). Scale bar 10 μm.

Human normal fibroblasts CCD-1070Sk after 72 h cultivation on CTRL substrate displayed normal adherence, spreading and morphology with cell line specificity. Phalloidin staining showed a normal phenotypic cytoskeleton organisation, with well-defined elongated actin filaments organised along the major axis of the cells and central nuclear display (Figure 11). A decrease in cell density was observed when fibroblast cells were grown on a PDMS scaffold, with results in line with that detected by MTS assay. Reduced bodies with different cytoskeleton distribution, with dotted-like focal contacts and peripheral nucleus localisation, can be seen on this type of substrate. In the case of MPC-coated PDMS scaffolds, cells recorded a different behaviour regarding spreading and morphology. Thus, induced cell elongation detected by polymerisation of actin fibrils but with a thinner cell body as compared with CTRL was observed. MPC addition determined a change in cell morphology, namely that the fibroblast cells modified the flatten appearance previously observed on CTRL and PDMS scaffolds, displaying a 3D organisation of the cell body and reducing the contact with material surface (Figure 10). SEM examination performed after 72 h of culture in direct contact with the scaffolds supported fluorescence microscopy results.

Using mouse fibroblasts L929 adherent cells in their study, Xu et al. have reported that SiO_2_ surfaces coated with MPC copolymers exhibit no cell adhesion after 1 to 4 days of culture [51]. A change in cell morphology, together with a reduced attachment and slow proliferation, was reported for human fibroblast KMST cells grown on MPC. Thus, typical fibroblastic spindle-shaped morphology changing into round-shaped with a large number of filopodia was reported [52].

The interaction of both cell lines, macrophages and fibroblasts, with PDMS–pMPC substrates which exhibit the highest hydrophilicity degree of all analysed materials as revealed by CA, and XPS measurements, resulted in a significant decrease in cell attachment and proliferation, accompanied by changes in cell morphology. Additionally, the newly modified PDMS, due to its characteristics, exhibited a strong resistance to bacterial colonisation and biofilm formation (Figure 9). It has been reported that hydrophilic coatings allow fewer inflammatory cells and bacteria to adhere on the surfaces, thus avoiding surface-induced undesired biological reactions such as inflammation, infection and fibrosis [56,57,58,59]. In our experiment, the moderate hydrophilic MPC coatings obtained by MAPLE technique showed better anti-cell and anti-bacterial adhesion capacity compared to hydrophobic PDMS. Moreover, the resulting PDMS–pMPC scaffolds show the advantage of having a stable surface chemistry and retain the hydrophilic character for a long time.

## 4. Conclusions

The MAPLE technique was used to coat a hydrophobic surface of Polydimethylsiloxane (PDMS) with a poly(2-Methacryloyloxyethyl-Phosphorylcholine) (pMPC) polymer layer targeting a hydrophilic interface for a controlled adherence of mammalian cells and microorganisms on the modified PDMS surfaces. The physical-chemical features investigation of the PDMS–pMPC interfaces revealed no significant alteration in the chemistry of the surfaces, accompanied by a decrease in the hydrophobicity characteristic of unmodified PDMS, a property maintained over two months.

Compared to chemical methods, the MAPLE technique proposed by us for the functionalisation of PDMS surface with pMPC has the advantage of achieving uniform and homogeneous moderate hydrophilic pMPC thin layers onto hydrophobic PDMS without any pre-treatment, therefore avoiding the major disadvantage of hydrophobicity recovery.

Biological investigation performed in vitro revealed a significantly reduced adherence on PDMS-MPC of both *E. coli* (from 29 × 10^6^ to 3 × 10^2^ CFU/mL) and *S. aureus* (from 29 × 10^6^ to 3 × 10^2^ CFU/mL) bacterial strains. Additionally, coated surfaces induced a significant inhibition of biofilm formation, an effect observed mainly for *E. coli.* Moreover, the pMPC coatings improved the capacity of PDMS to reduce the adhesion and proliferation of human THP-1 differentiated macrophages cells by 50%, and of CCD-1070Sk human normal fibroblasts by 40%, compared to the unmodified scaffold, thus circumventing undesired cell responses such as inflammation and fibrosis.

All this highlighted the potential for the newly PDMS–pMPC scaffolds obtained by MAPLE to be used for better mitigating microbial and foreign body response.

## Data Availability

Not applicable.

## Supplementary Materials

The following supporting information can be downloaded at: https://www.mdpi.com/article/10.3390/nano13010064/s1, Figure S1. (A) Atomic force microscopy images of pMPC deposited by MAPLE at 250 mJ/cm^2^ onto PDMS, with the root mean square roughness (Rq) of 65 nm for a scan area of 40 × 40 μm^2^. (B) SEM images of pMPC deposited at 250 mJ/cm^2^ on untreated PDMS; Figure S2. SEM images of pMPC deposited at 450 mJ/cm2 on UV Ozone treated PDMS; Figure S3. XPS comparison spectra of pMPC 2 wt.% on Si: drop-cast (control) (a) XPS full spectra (survey), (b) C1s high resolution spectra with (c) XPS full spectra (coloured survey) of Si-pMPC interfaces deposited by MAPLE method at different laser fluences, and (d) C1s spectra of Si-pMPC interface at 450 mJ/cm^2^. Figure S4. XPS high resolution spectra comparison of the pMPC 2 wt.% on Si: drop-cast pMPC (a) N1s spectra, and (b) P2p spectra, and PDMS–pMPC interfaces deposited by MAPLE at 450 mJ/cm^2^; (c) N1s spectra, (d) P2p spectra; Figure S5. Contact angle and surface energy measurements of pMPC coatings obtained by MAPLE on Si; Figure S6. Histograms of contact angle and surface energy measurements onto PDMS and pMPC coatings onto treated PDMS; Figure S7. Histograms of contact angle onto PDMS–pMPC coatings onto treated PDMS after 2 months; Figure S8. SEM images of pMPC coatings deposited on PDMS after immersion in water. Although the surface suffered modifications, and disappearance of the microglobular structures is characteristic in comparison with that of before the water treatment, all the pMPC were found to be on PDMS; Figure S9. Indirect contact experiments, THP-1 derived macrophages exposed to extracts obtained by direct incubation of scaffold materials PDMS and PDMS-pMPC with cell medium. Micrographs of whole area of the sample and inset of relevant field of view 10× magnification (scale bar = 100 µm) of THP-1 derived macrophages’ morphology in the absence (A) or presence of LPS (B). Metabolic activity of THP-1-derived macrophages (C) in indirect contact experiments with or without endotoxin stimulation. Cells cultured on glass coverslip and incubated with cell medium served as control (CTRL); Figure S10. Indirect contact experiments, CCD1070-Sk fibroblasts exposed to extracts obtained by direct incubation of scaffold materials PDMS and PDMS-pMPC with cell medium. Micrographs of whole area of the sample and inset of relevant field of view 10× magnification (scale bar = 100 µm) of fibroblasts’ morphology (A). Metabolic activity of CCD1070-Sk fibroblasts in indirect contact experiments after 3 days of culture (B). Cells cultured on glass coverslip and incubated with cell medium served as control (CTRL).
