# Supplementary material for: Mitigation of Cellular and Bacterial Adhesion on Laser Modified Poly (2-Methacryloyloxyethyl Phosphorylcholine)/Polydimethylsiloxane Surface
Source: Nanomaterials (Basel). 2022 Dec 23;13(1):64. doi: 10.3390/nano13010064 (PMC9824587; doi:10.3390/nano13010064)
Supplement: Supplementary file 1 [file nanomaterials-13-00064-s001.zip › nanomaterials-2070197-supplementary.pdf]

Article

Mitigation of Cellular and Bacterial Adhesion on Laser Modified Poly (2-Methacryloyloxyethyl Phosphorylcholine)/Polydimethylsiloxane Surface

Simona Nistorescu [1,2,†], Madalina Icriverzi [3,†], Paula Florian [3,†], Anca Bonciu [1], Valentina Marascu [1], Nicoleta Dumitrescu [1], Gratiela Gradisteanu Pircalabioru [4], Laurentiu Rusen [1], Alexandra Mocanu [5], Anca Roseanu [3], Anisoara Cimpean [2], Florin Grama [6,*], Valentina Dinca [1,*] and Daniel A. Cristian [6]

[1] National Institute for Laser, Plasma and Radiation Physics, 077125 Magurele, Romania
[2] Faculty of Biology, University of Bucharest, 050095 Bucharest, Romania
[3] Institute for Biochemistry of Romanian Academy, 060031, Bucharest, Romania
[4] Research Institute of the University of Bucharest, 050095 Bucharest, Romania
[5] University Politehnica of Bucharest, Faculty of Chemical Engineering and Biotechnologies, 011061 Bucharest, Romania
[6] Surgical Department, "Carol Davila" University of Medicine and Pharmacy, 030171 Bucharest, Romania
* Correspondence: florin.grama@umfcd.ro (F.G.); valentina.dinca@inflpr.ro (V.D.); Tel.: +40-214574414 (V.D.)
† Those authors contributed equally to this manuscript.

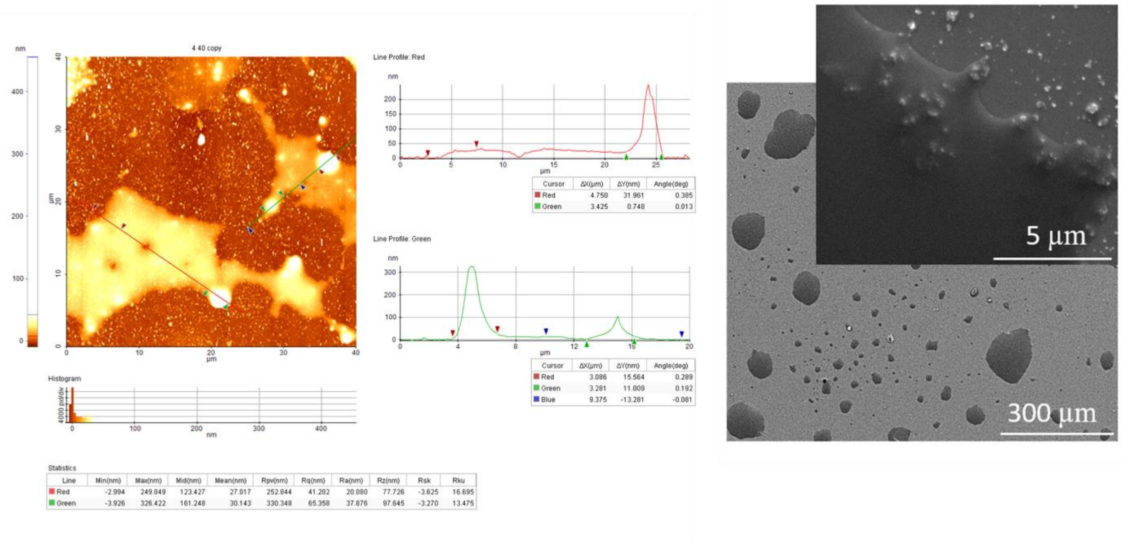

Figure S1. A) Atomic force microscopy images of pMPC deposited by MAPLE at 250 mJ/cm^2 onto PDMS, with the root mean square roughness (Rq) of 65 nm for a scan area of 40 × 40 μm^2.B) SEM images of pMPC deposited at 250 mJ/cm2 on untreated PDMS

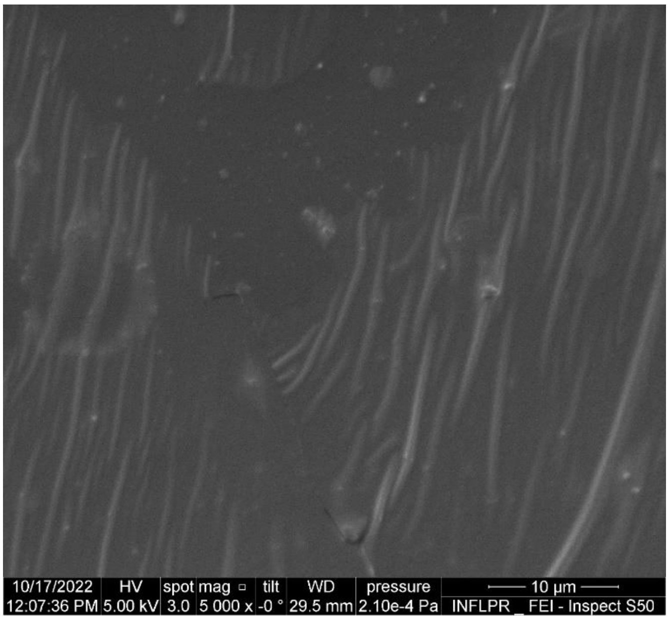

Figure S2. SEM images of pMPC deposited at 450 mJ/cm2 on UV Ozone treated PDMS

A B

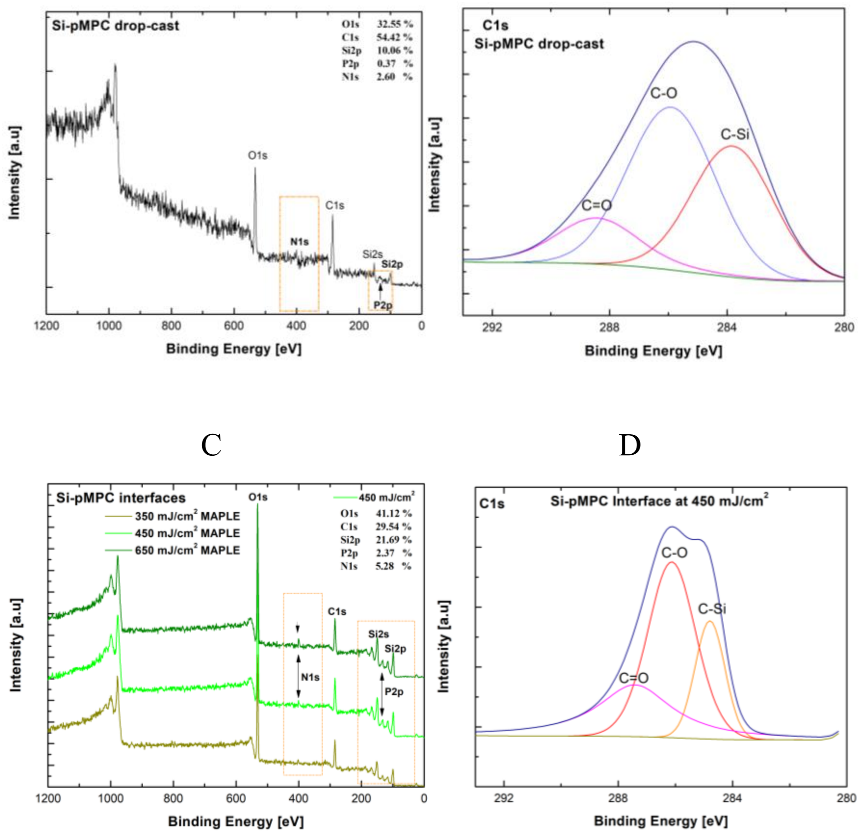

Figure S3. XPS comparison spectra's of pMPC 2 wt.% on Si: drop-cast (control) (A) XPS full spectra (survey), (B) C1s high resolution spectra with (C) XPS full spectra (coloured survey) of Si-pMPC interfaces deposited by MAPLE method at different laser fluences, and (D) C1s spectra of Si-pMPC interface at 450 mJ/cm^2

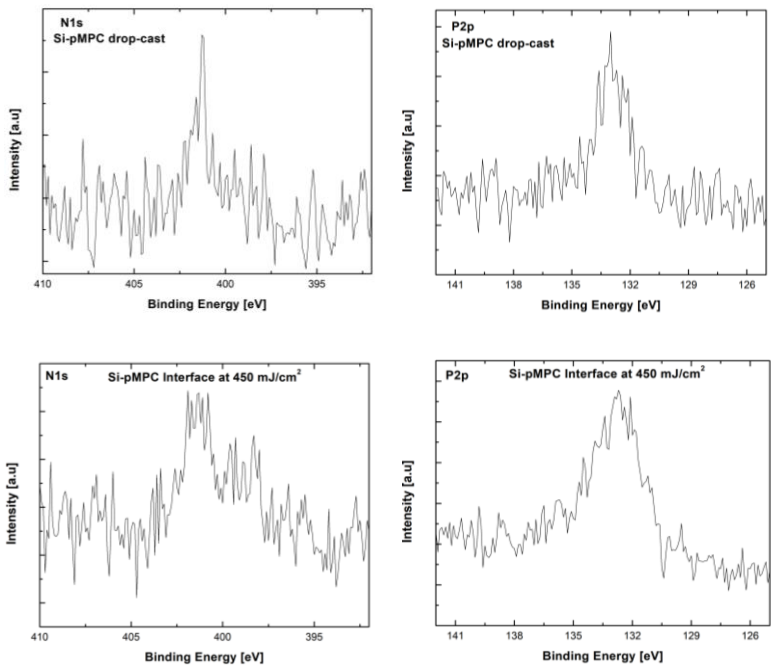

Figure S4. XPS high resolution spectra comparison of the pMPC 2 wt.% on Si: drop-cast pMPC (a) N1s spectra, and (b) P2p spectra, and PDMS-pMPC interfaces deposited by MAPLE at 450mJ/cm^2; (c) N1s spectra, (d) P2p spectra

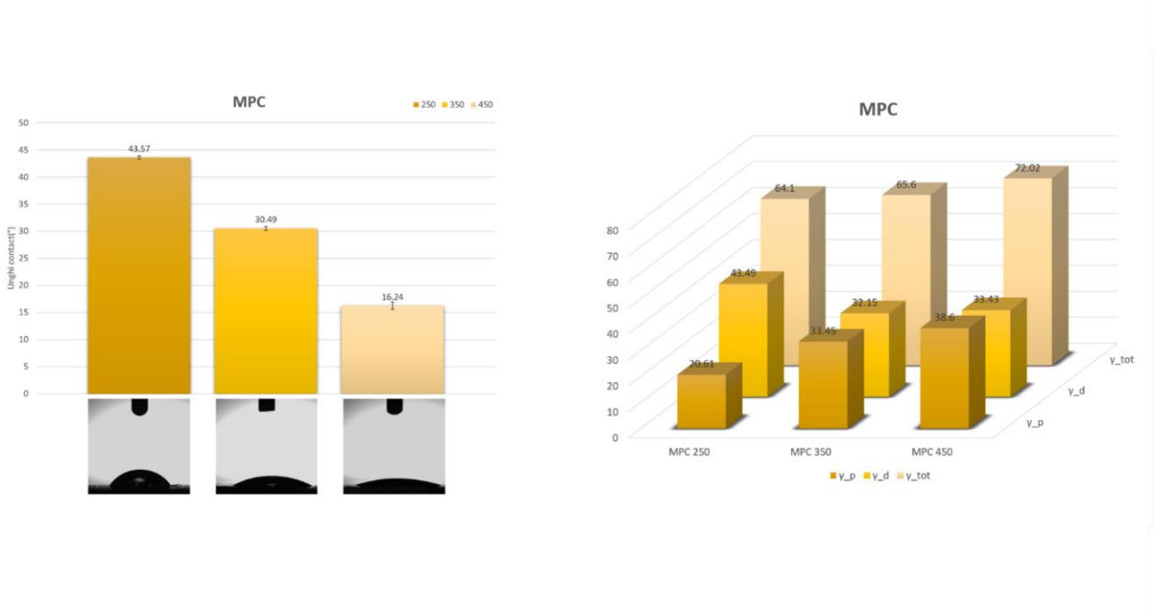

Figure S5. Contact angle and surface energy measurements of pMPC coatings obtained by MAPLE on Si

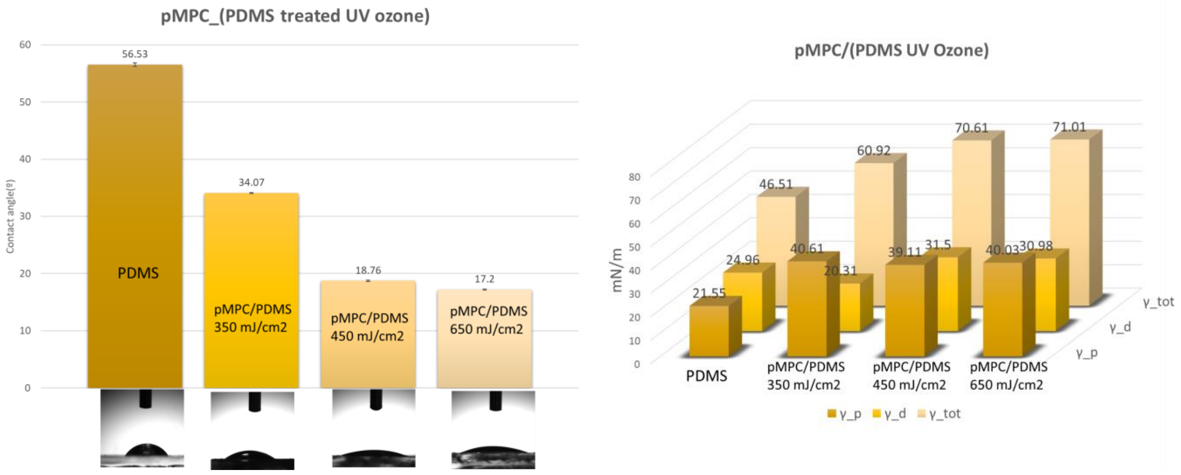

Figure S6. Histograms of contact angle and surface energy measurements onto PDMS and pMPC coatings onto treated PDMS

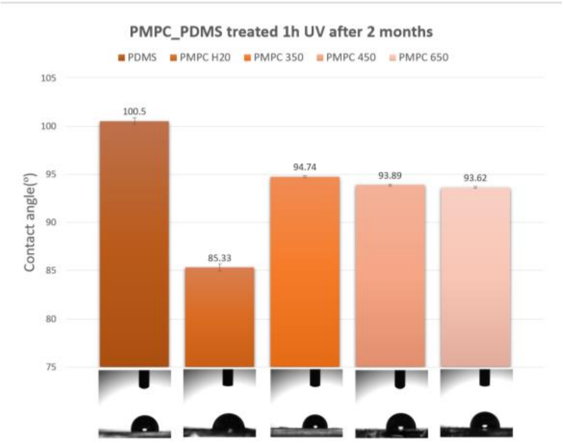

Figure S7. Histograms of contact angle onto PDMS-pMPC coatings onto treated PDMS after 2 months

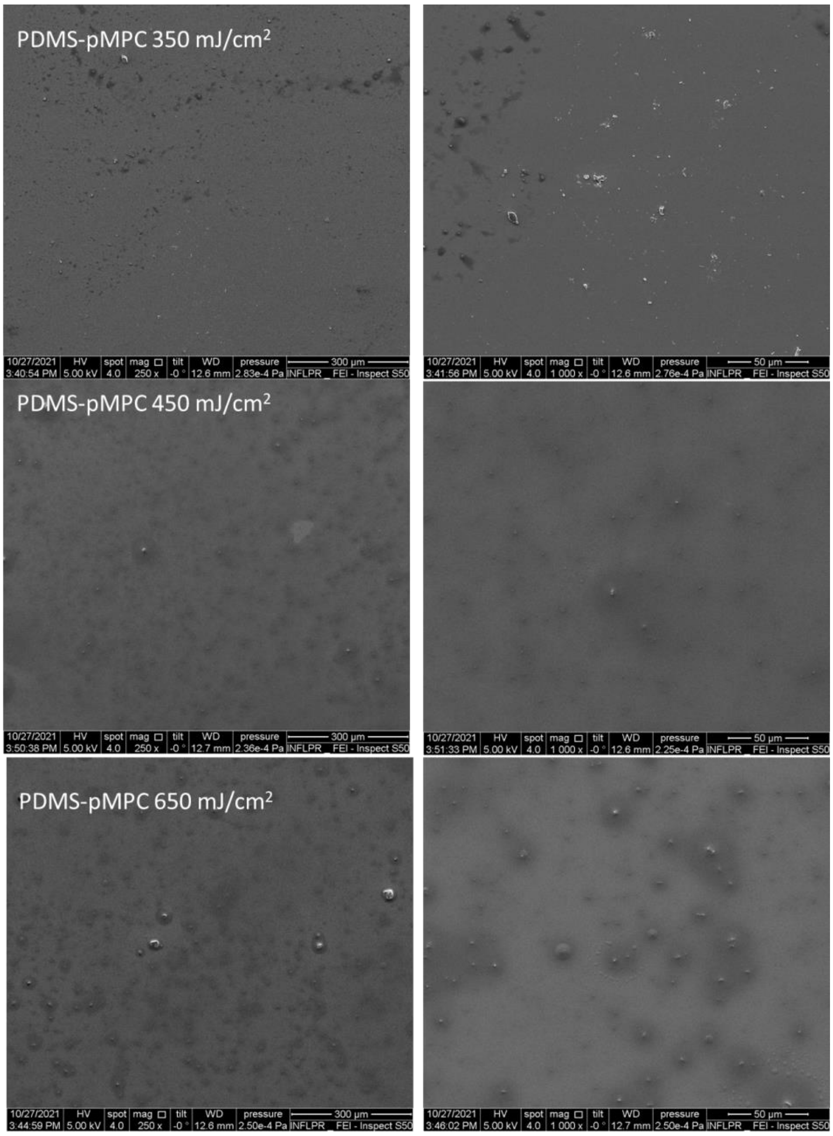

Figure S8. SEM images of pMPC coatings deposited on PDMS after immersion in water. Although the surface suffered modifications, and disappearance of the microglobular structures is characteristic in comparison with that of before the water treatment, the coating of pMPC were found to be on PDMS.

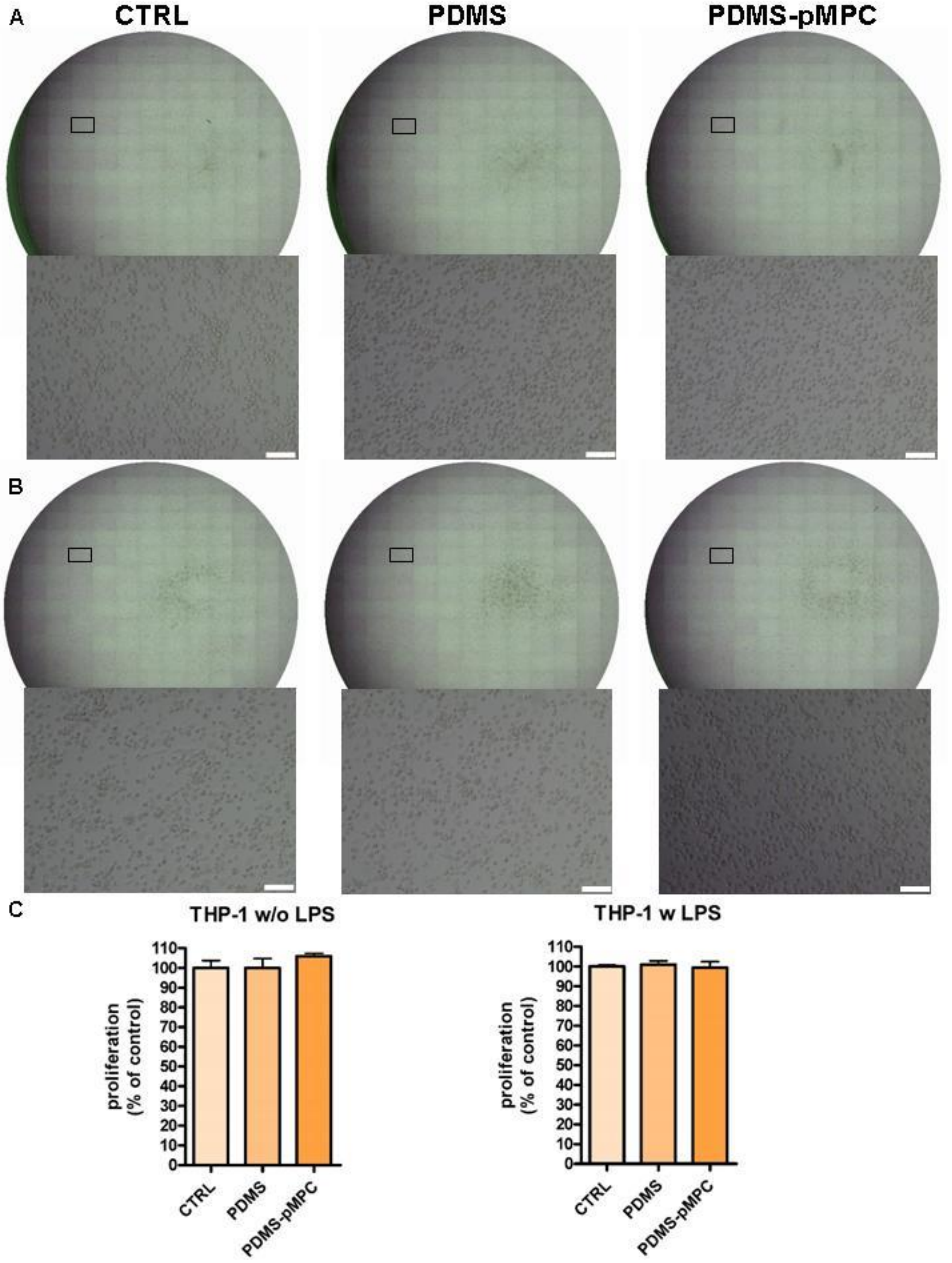

Figure S9. Indirect contact experiments, THP-1 derived macrophages exposed to extracts obtained by direct incubation of scaffold materials PDMS and PDMS-pMPC with cell medium. Micrographs of whole area of the sample and inset of relevant field of view 10× magnification (scale bar = 100 μm) of THP-1 derived macrophages' morphology in the absence

(A) or presence of LPS (B). Metabolic activity of THP-1-derived macrophages (C) in indirect contact experiments with or without endotoxin stimulation. Cells cultured on glass coverslip and incubated with cell medium served as control (CTRL).

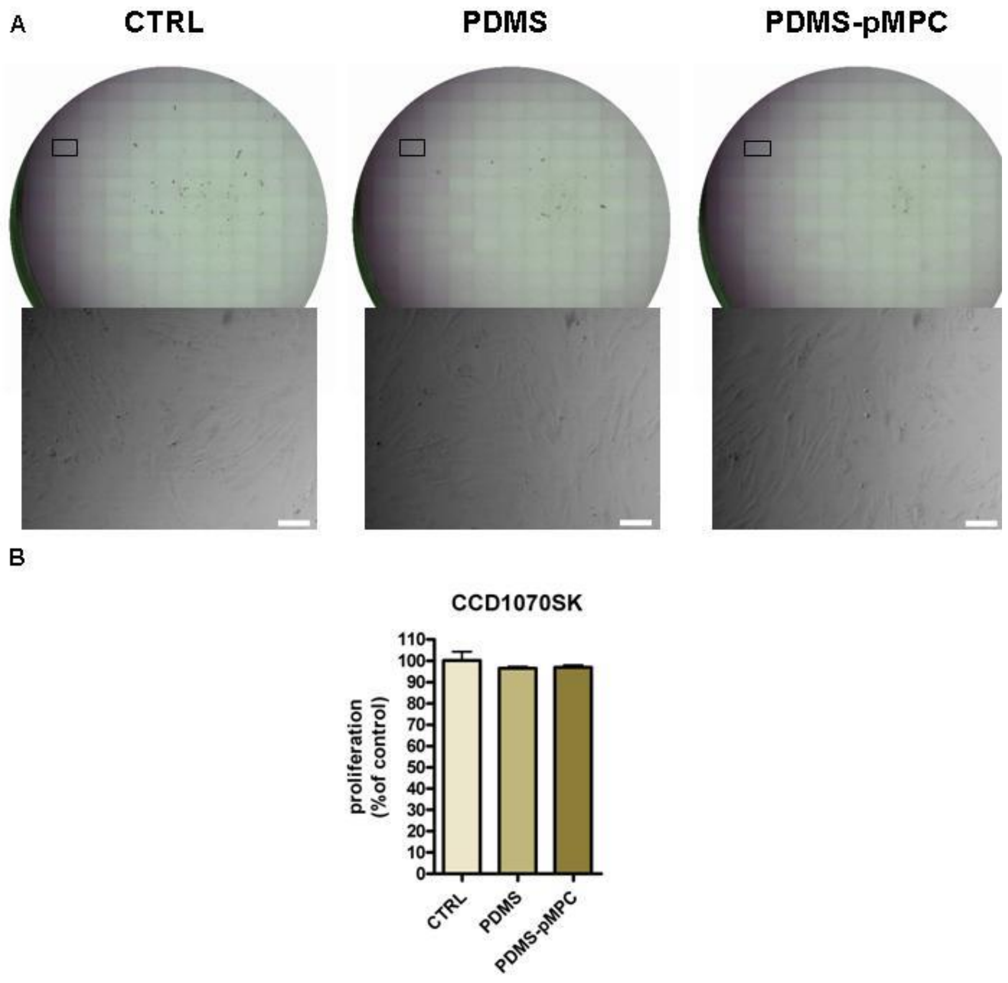

Figure S10. Indirect contact experiments, CCD1070-Sk fibroblasts exposed to extracts obtained by direct incubation of scaffold materials PDMS and PDMS-pMPC with cell medium. Micrographs of whole area of the sample and inset of relevant field of view 10× magnification (scale bar = 100 μm) of fibroblasts' morphology (A). Metabolic activity of CCD1070-Sk fibroblasts in indirect contact experiments after 3 days of culture (B). Cells cultured on glass coverslip and incubated with cell medium served as control (CTRL)
